# *N*′-(2-Hydr­oxy-5-nitro­benzyl­idene)benzene­sulfonohydrazide

**DOI:** 10.1107/S1600536808022691

**Published:** 2008-07-23

**Authors:** Juahir Yusnita, Hapipah M. Ali, Subramaniam Puvaneswary, Ward T. Robinson, Seik Weng Ng

**Affiliations:** a Department of Chemistry, University of Malaya, 50603 Kuala Lumpur, Malaysia

## Abstract

The mol­ecule of the title compound, C_13_H_11_N_3_O_5_S, shows a phenyl group and an almost planar intra­molecularly hydrogen-bonded *N*′-(2-hydr­oxy-5-phenyl­ebenzyl­idene)hydrazino group disposed about the S atom. Adjacent mol­ecules are linked by N—H⋯O_nitro_ hydrogen bonds, producing a linear chain that runs along the *b* axis of the unit cell.

## Related literature

For 2′-[1-(2-hydroxy­phen­yl)ethyl­idene]benzene­sulfono­hydra­zide, see: Tai *et al.* (2008 ▶).
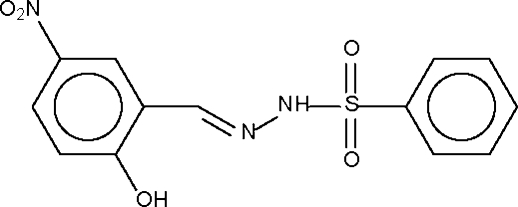


## Experimental

### 

#### Crystal data

C_13_H_11_N_3_O_5_S
*M* *_r_* = 321.31
Monoclinic, 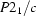

*a* = 7.8188 (1) Å
*b* = 14.5904 (2) Å
*c* = 11.9083 (1) Å
β = 98.159 (1)°
*V* = 1344.74 (3) Å^3^
*Z* = 4
Mo *K*α radiation
μ = 0.27 mm^−1^
*T* = 100 (2) K
0.47 × 0.40 × 0.33 mm

#### Data collection

Bruker SMART APEX diffractometer
Absorption correction: multi-scan (*SADABS*; Sheldrick, 1996 ▶) *T*_min_ = 0.883, *T*_max_ = 0.916
16761 measured reflections
3089 independent reflections
2972 reflections with *I* > 2σ(*I*)
*R*_int_ = 0.024

#### Refinement

*R*[*F* ^2^ > 2σ(*F* ^2^)] = 0.032
*wR*(*F* ^2^) = 0.088
*S* = 1.03
3089 reflections
243 parameters
11 restraints
H atoms treated by a mixture of independent and constrained refinement
Δρ_max_ = 0.42 e Å^−3^
Δρ_min_ = −0.51 e Å^−3^

### 

Data collection: *APEX2* (Bruker, 2007 ▶); cell refinement: *SAINT* (Bruker, 2007 ▶); data reduction: *SAINT*; program(s) used to solve structure: *SHELXS97* (Sheldrick, 2008 ▶); program(s) used to refine structure: *SHELXL97* (Sheldrick, 2008 ▶); molecular graphics: *X-SEED* (Barbour, 2001 ▶); software used to prepare material for publication: *publCIF* (Westrip, 2008 ▶).

## Supplementary Material

Crystal structure: contains datablocks global, I. DOI: 10.1107/S1600536808022691/im2077sup1.cif

Structure factors: contains datablocks I. DOI: 10.1107/S1600536808022691/im2077Isup2.hkl

Additional supplementary materials:  crystallographic information; 3D view; checkCIF report

## Figures and Tables

**Table 1:** Hydrogen-bond geometry (Å, °)

*D*—H⋯*A*	*D*—H	H⋯*A*	*D*⋯*A*	*D*—H⋯*A*
O1—H1*o*⋯N2	0.84 (1)	1.86 (1)	2.628 (1)	153 (2)
N3—H3*n*⋯O3^i^	0.88 (1)	2.13 (1)	2.978 (1)	163 (2)

Symmetry code: (i) 
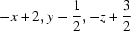
.

**Fig. 1.**
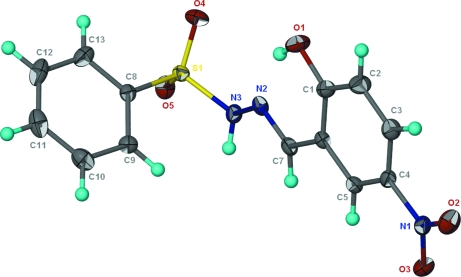
Thermal ellipsoid plot (Barbour, 2001) plot of C13H11N3O5S at the 70% probability level. Hydrogen atoms are drawn as spheres of arbitrary radius.

**Fig. 2.**
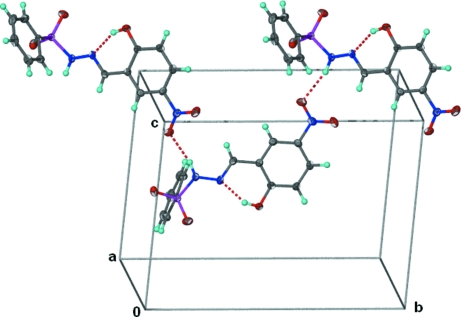
Hydrogen-bonded chain structure. Hydrogen bonds are denoted by dashed lines.

## supplementary crystallographic information

### Comment

The molecules of benzenesulfonic acid-(2-hydroxy-5-nitro-benzylidene)-hydrazide adopt a linear chain-like structure in which the individual molecules are linked by N–H···O hydrogen bonds [3.093 (3) Å] (Tai *et al.*, 2008). The title compound exhibits a phenyl group and an almost planar, intramolecularly hydrogen-bonded (2-hydroxy-5-phenyl)ethylidenehydrazidyl group disposed about the sulfur atom. The nitro group is situated *para* with respect to the OH donor site (Scheme I, Fig. 1). The nitro group also acts as an acceptor towards the amino group of an adjacent molecule to furnish a chain-like structure that runs along the *b*-axis of the unit cell (Fig. 2).

### Experimental

2-Hydroxy-5-nitrobenzaldehyde (0.50 g, 3 mmol) and benzene sulfonylhydrazide (0.52 g, 0.3 mmol) were condensed in refluxing ethanol (100 ml) for two hours. The solvent was removed to give the Schiff base, which was collected in nearly quantative yield. and dried. Crystals were obtained by recrystallization from ethanol.

### Refinement

All hydrogen atoms were located in a difference Fouier map, and were refined with distance restraints of C–H 0.95±0.01, N–H 0.88±0.01 and O–H 0.84±0.01 Å. Their temperature factors were refined freely.

### Crystal data

**Table:**

C_13_H_11_N_3_O_5_S	*F*_000_ = 664
*M**_r_* = 321.31	*D*_x_ = 1.587 Mg m^−^^3^
Monoclinic, *P*2_1_/*c*	Mo *K*α radiation λ = 0.71073 Å
Hall symbol: -P 2ybc	Cell parameters from 9991 reflections
*a* = 7.8188 (1) Å	θ = 2.4–30.4º
*b* = 14.5904 (2) Å	µ = 0.27 mm^−^^1^
*c* = 11.9083 (1) Å	*T* = 100 (2) K
β = 98.159 (1)º	Triangular plate, yellow
*V* = 1344.74 (3) Å^3^	0.47 × 0.40 × 0.33 mm
*Z* = 4	

### Data collection

**Table:**

Bruker SMART APEX diffractometer	3089 independent reflections
Radiation source: fine-focus sealed tube	2972 reflections with *I* > 2σ(*I*)
Monochromator: graphite	*R*_int_ = 0.024
*T* = 100(2) K	θ_max_ = 27.5º
ω scans	θ_min_ = 2.2º
Absorption correction: Multi-scan(SADABS; Sheldrick, 1996)	*h* = −10→9
*T*_min_ = 0.883, *T*_max_ = 0.916	*k* = −18→18
16761 measured reflections	*l* = −14→15

### Refinement

**Table:**

Refinement on *F*^2^	Secondary atom site location: difference Fourier map
Least-squares matrix: full	Hydrogen site location: inferred from neighbouring sites
*R*[*F*^2^ > 2σ(*F*^2^)] = 0.032	H atoms treated by a mixture of independent and constrained refinement
*wR*(*F*^2^) = 0.088	*w* = 1/[σ^2^(*F*_o_^2^) + (0.0521*P*)^2^ + 0.7306*P*] where *P* = (*F*_o_^2^ + 2*F*_c_^2^)/3
*S* = 1.04	(Δ/σ)_max_ = 0.001
3089 reflections	Δρ_max_ = 0.42 e Å^−^^3^
243 parameters	Δρ_min_ = −0.51 e Å^−^^3^
11 restraints	Extinction correction: none
Primary atom site location: structure-invariant direct methods	

### Fractional atomic coordinates and isotropic or equivalent isotropic displacement parameters (Å^2^)

**Table:**

	*x*	*y*	*z*	*U*_iso_*/*U*_eq_	
S1	0.78969 (4)	0.172790 (19)	0.37657 (2)	0.01358 (10)	
N1	0.89489 (14)	0.67461 (7)	0.75588 (9)	0.0169 (2)	
N2	0.82479 (13)	0.33431 (7)	0.46383 (9)	0.0149 (2)	
N3	0.89849 (13)	0.24787 (7)	0.46141 (9)	0.0150 (2)	
O1	0.65151 (13)	0.47538 (6)	0.36923 (8)	0.0233 (2)	
O2	0.84138 (13)	0.75301 (6)	0.76435 (8)	0.0239 (2)	
O3	1.00400 (12)	0.63904 (7)	0.82771 (8)	0.0224 (2)	
O4	0.74085 (13)	0.21585 (7)	0.26881 (7)	0.0230 (2)	
O5	0.89964 (11)	0.09391 (6)	0.38421 (8)	0.01914 (19)	
C1	0.70881 (16)	0.52031 (8)	0.46569 (10)	0.0166 (2)	
C2	0.66093 (17)	0.61234 (9)	0.47357 (11)	0.0202 (3)	
C3	0.71928 (16)	0.66280 (8)	0.56918 (11)	0.0181 (2)	
C4	0.82690 (15)	0.62056 (8)	0.65697 (10)	0.0150 (2)	
C5	0.87528 (15)	0.52972 (8)	0.65268 (10)	0.0146 (2)	
C6	0.81634 (15)	0.47794 (8)	0.55664 (10)	0.0143 (2)	
C7	0.87482 (15)	0.38342 (8)	0.55157 (10)	0.0149 (2)	
C8	0.60116 (14)	0.14633 (8)	0.43497 (10)	0.0129 (2)	
C9	0.58974 (16)	0.16378 (8)	0.54832 (10)	0.0157 (2)	
C10	0.44273 (17)	0.13509 (9)	0.59181 (11)	0.0208 (3)	
C11	0.31115 (16)	0.08982 (9)	0.52296 (13)	0.0231 (3)	
C12	0.32481 (16)	0.07308 (9)	0.41038 (13)	0.0231 (3)	
C13	0.47021 (16)	0.10120 (9)	0.36497 (11)	0.0189 (2)	
H1O	0.692 (3)	0.4223 (8)	0.3795 (18)	0.043 (6)*	
H3N	0.944 (2)	0.2249 (12)	0.5268 (10)	0.027 (4)*	
H2	0.587 (2)	0.6401 (12)	0.4127 (12)	0.028 (4)*	
H3	0.691 (2)	0.7249 (7)	0.5788 (15)	0.025 (4)*	
H5	0.9475 (18)	0.5022 (10)	0.7134 (11)	0.019 (4)*	
H7	0.9524 (18)	0.3611 (11)	0.6142 (10)	0.019 (4)*	
H9	0.6803 (17)	0.1930 (11)	0.5961 (12)	0.023 (4)*	
H10	0.431 (2)	0.1463 (13)	0.6687 (9)	0.033 (5)*	
H11	0.2133 (16)	0.0685 (11)	0.5530 (14)	0.026 (4)*	
H12	0.2367 (19)	0.0410 (11)	0.3635 (13)	0.030 (4)*	
H13	0.482 (2)	0.0875 (11)	0.2882 (9)	0.025 (4)*	

### Atomic displacement parameters (Å^2^)

**Table:**

	*U*^11^	*U*^22^	*U*^33^	*U*^12^	*U*^13^	*U*^23^
S1	0.01629 (16)	0.01318 (16)	0.01162 (15)	−0.00058 (9)	0.00324 (11)	−0.00033 (9)
N1	0.0174 (5)	0.0180 (5)	0.0160 (5)	−0.0050 (4)	0.0042 (4)	−0.0018 (4)
N2	0.0170 (5)	0.0106 (4)	0.0175 (5)	−0.0002 (3)	0.0038 (4)	0.0021 (3)
N3	0.0161 (5)	0.0110 (4)	0.0174 (5)	−0.0007 (4)	0.0006 (4)	0.0002 (4)
O1	0.0356 (5)	0.0154 (4)	0.0158 (4)	0.0037 (4)	−0.0069 (4)	−0.0016 (3)
O2	0.0272 (5)	0.0183 (4)	0.0260 (5)	−0.0003 (4)	0.0035 (4)	−0.0078 (4)
O3	0.0245 (5)	0.0236 (5)	0.0174 (4)	−0.0045 (4)	−0.0028 (4)	−0.0012 (3)
O4	0.0299 (5)	0.0266 (5)	0.0125 (4)	−0.0012 (4)	0.0028 (4)	0.0040 (3)
O5	0.0187 (4)	0.0158 (4)	0.0240 (5)	0.0009 (3)	0.0067 (3)	−0.0037 (3)
C1	0.0203 (6)	0.0150 (6)	0.0139 (5)	−0.0006 (4)	0.0003 (4)	0.0002 (4)
C2	0.0250 (6)	0.0160 (6)	0.0180 (6)	0.0030 (5)	−0.0024 (5)	0.0018 (4)
C3	0.0205 (6)	0.0133 (5)	0.0204 (6)	0.0012 (4)	0.0026 (5)	0.0000 (4)
C4	0.0162 (5)	0.0153 (5)	0.0137 (5)	−0.0037 (4)	0.0031 (4)	−0.0018 (4)
C5	0.0149 (5)	0.0158 (5)	0.0132 (5)	−0.0022 (4)	0.0020 (4)	0.0023 (4)
C6	0.0156 (5)	0.0132 (5)	0.0142 (5)	−0.0016 (4)	0.0028 (4)	0.0018 (4)
C7	0.0156 (5)	0.0136 (5)	0.0158 (5)	−0.0012 (4)	0.0027 (4)	0.0034 (4)
C8	0.0132 (5)	0.0109 (5)	0.0148 (5)	0.0008 (4)	0.0023 (4)	0.0007 (4)
C9	0.0162 (5)	0.0163 (5)	0.0147 (5)	0.0007 (4)	0.0020 (4)	0.0001 (4)
C10	0.0210 (6)	0.0215 (6)	0.0214 (6)	0.0038 (5)	0.0084 (5)	0.0036 (5)
C11	0.0156 (6)	0.0169 (6)	0.0383 (8)	0.0020 (4)	0.0090 (5)	0.0046 (5)
C12	0.0154 (6)	0.0164 (6)	0.0359 (7)	−0.0010 (4)	−0.0020 (5)	−0.0038 (5)
C13	0.0183 (6)	0.0172 (6)	0.0199 (6)	0.0012 (4)	−0.0020 (4)	−0.0036 (4)

### Geometric parameters (Å, °)

**Table:**

S1—O4	1.4312 (9)	C3—H3	0.943 (9)
S1—O5	1.4316 (9)	C4—C5	1.3812 (17)
S1—N3	1.6434 (10)	C5—C6	1.3934 (16)
S1—C8	1.7597 (12)	C5—H5	0.942 (9)
N1—O2	1.2271 (14)	C6—C7	1.4569 (16)
N1—O3	1.2336 (14)	C7—H7	0.950 (9)
N1—C4	1.4540 (15)	C8—C9	1.3889 (16)
N2—C7	1.2815 (16)	C8—C13	1.3908 (16)
N2—N3	1.3886 (13)	C9—C10	1.3905 (17)
N3—H3N	0.875 (9)	C9—H9	0.945 (9)
O1—C1	1.3431 (14)	C10—C11	1.3884 (19)
O1—H1O	0.838 (9)	C10—H10	0.947 (9)
C1—C2	1.4006 (17)	C11—C12	1.382 (2)
C1—C6	1.4155 (16)	C11—H11	0.942 (9)
C2—C3	1.3780 (17)	C12—C13	1.3884 (19)
C2—H2	0.952 (9)	C12—H12	0.947 (9)
C3—C4	1.3897 (17)	C13—H13	0.953 (9)
			
O4—S1—O5	119.43 (6)	C4—C5—H5	121.2 (10)
O4—S1—N3	107.91 (6)	C6—C5—H5	119.4 (10)
O5—S1—N3	104.06 (5)	C5—C6—C1	118.86 (11)
O4—S1—C8	108.59 (6)	C5—C6—C7	118.56 (10)
O5—S1—C8	109.12 (5)	C1—C6—C7	122.52 (11)
N3—S1—C8	107.03 (5)	N2—C7—C6	120.12 (11)
O2—N1—O3	123.06 (11)	N2—C7—H7	122.4 (10)
O2—N1—C4	118.84 (10)	C6—C7—H7	117.4 (10)
O3—N1—C4	118.09 (10)	C9—C8—C13	121.73 (11)
C7—N2—N3	116.43 (10)	C9—C8—S1	121.12 (9)
N2—N3—S1	115.98 (8)	C13—C8—S1	116.98 (9)
N2—N3—H3N	116.5 (12)	C8—C9—C10	118.51 (11)
S1—N3—H3N	113.7 (12)	C8—C9—H9	121.5 (10)
C1—O1—H1O	104.7 (15)	C10—C9—H9	120.0 (10)
O1—C1—C2	117.73 (10)	C11—C10—C9	120.38 (12)
O1—C1—C6	122.07 (11)	C11—C10—H10	119.2 (12)
C2—C1—C6	120.18 (11)	C9—C10—H10	120.4 (12)
C3—C2—C1	120.48 (11)	C12—C11—C10	120.28 (12)
C3—C2—H2	119.8 (11)	C12—C11—H11	119.3 (11)
C1—C2—H2	119.7 (11)	C10—C11—H11	120.4 (11)
C2—C3—C4	118.60 (11)	C11—C12—C13	120.40 (12)
C2—C3—H3	123.8 (11)	C11—C12—H12	120.7 (11)
C4—C3—H3	117.6 (11)	C13—C12—H12	118.9 (11)
C5—C4—C3	122.51 (11)	C12—C13—C8	118.71 (12)
C5—C4—N1	118.68 (10)	C12—C13—H13	120.5 (11)
C3—C4—N1	118.79 (11)	C8—C13—H13	120.8 (11)
C4—C5—C6	119.35 (11)		
			
C7—N2—N3—S1	158.80 (9)	O1—C1—C6—C7	1.05 (19)
O4—S1—N3—N2	47.89 (10)	C2—C1—C6—C7	−177.91 (11)
O5—S1—N3—N2	175.72 (8)	N3—N2—C7—C6	174.40 (10)
C8—S1—N3—N2	−68.82 (9)	C5—C6—C7—N2	−179.10 (11)
O1—C1—C2—C3	−178.31 (12)	C1—C6—C7—N2	−2.18 (18)
C6—C1—C2—C3	0.69 (19)	O4—S1—C8—C9	−135.67 (10)
C1—C2—C3—C4	0.3 (2)	O5—S1—C8—C9	92.63 (10)
C2—C3—C4—C5	−1.02 (19)	N3—S1—C8—C9	−19.41 (11)
C2—C3—C4—N1	177.34 (11)	O4—S1—C8—C13	48.99 (11)
O2—N1—C4—C5	−174.10 (11)	O5—S1—C8—C13	−82.72 (10)
O3—N1—C4—C5	5.76 (16)	N3—S1—C8—C13	165.25 (9)
O2—N1—C4—C3	7.48 (17)	C13—C8—C9—C10	−0.06 (18)
O3—N1—C4—C3	−172.65 (11)	S1—C8—C9—C10	−175.19 (9)
C3—C4—C5—C6	0.71 (18)	C8—C9—C10—C11	0.18 (18)
N1—C4—C5—C6	−177.64 (10)	C9—C10—C11—C12	−0.20 (19)
C4—C5—C6—C1	0.30 (17)	C10—C11—C12—C13	0.1 (2)
C4—C5—C6—C7	177.34 (10)	C11—C12—C13—C8	0.03 (19)
O1—C1—C6—C5	177.97 (11)	C9—C8—C13—C12	−0.04 (18)
C2—C1—C6—C5	−0.99 (18)	S1—C8—C13—C12	175.28 (9)

### Hydrogen-bond geometry (Å, °)

**Table:**

*D*—H···*A*	*D*—H	H···*A*	*D*···*A*	*D*—H···*A*
O1—H1o···N2	0.84 (1)	1.86 (1)	2.628 (1)	153 (2)
N3—H3n···O3^i^	0.88 (1)	2.13 (1)	2.978 (1)	163 (2)

Symmetry codes: (i) −*x*+2, *y*−1/2, −*z*+3/2.
